# The Effectiveness of the Pitstop Method of Teaching Among Phase II Medical Students

**DOI:** 10.7759/cureus.71906

**Published:** 2024-10-20

**Authors:** Subalakshmi Balasubramanian, Sreekumar EJ, Nellaiyappan Balasubramanian, Mohanapriya Thyagarajan, Shanthi Mariappan, Sandhya Sundaram, K Balaji Singh

**Affiliations:** 1 Pathology, Sri Ramachandra Institute of Higher Education and Research, Chennai, IND; 2 Anaesthesiology, Sri Ramachandra Institute of Higher Education and Research, Chennai, IND; 3 Hand Surgery, Sri Ramachandra Institute of Higher Education and Research, Chennai, IND; 4 General Surgery, Sri Ramachandra Institute of Higher Education and Research, Chennai, IND; 5 Microbiology, Sri Ramachandra Institute of Higher Education and Research, Chennai, IND; 6 Surgery, Sri Ramachandra Institute of Higher Education and Research, Chennai, IND

**Keywords:** attention, flashcard, innovative teaching method, pitstop method, shortvideo

## Abstract

Background and objectives

The pitstop method of teaching involves short educational videos that entail a stepwise approach to augment knowledge retention and understanding. Short videos stimulate both visual and auditory pathways, thereby improving learner’s experiences, knowledge retention, and understanding of the concept, which will help with long-term memory. In this study, we aimed to compare the pitstop method of teaching with the didactic lecture technique based on assessment and feedback.

Methodology

After obtaining the Institutional Ethics Committee (IEC) approval, a comparative cross-sectional study was conducted among 210 phase II MBBS students who gave consent to participate in the study. Two sessions comprising a total of 10 videos with five videos in each session were planned for a particular system. Two types of teaching methodology were used in the videos. For session 1, a short video using a presentation template was used. For session 2, a short video using hand notes and figures was prepared and incorporated into the video. Each video was two minutes in duration. Students were divided into A and B batches. For session 1, A batch was the control group and B batch was the test group; for session 2, the test and control groups were reversed. Pre-test and post-test exercises were conducted for each session. Results were analyzed and statistical analysis was performed at the end of the project.

Results

An analysis of both sessions was done for the A and B batches. For session 1, the test group fared well with a mean of 4.50 compared to the control group with a mean of 3.53. For session 2, the test group performed well with a mean of 4.43 compared to the control group with a mean of 3.87. No significant difference was observed when the test groups of the two sessions were compared (p=0.74).

Conclusions

Short videos delivered by using the pitstop method will help stimulate memory and enhance the attention of students to better process the information. We believe that the implementation of this method will lead to good outcomes among the students.

## Introduction

In today's world, we are rapidly stepping into an age where information is delivered in a fragmented way. A short video in its traditional sense refers to any video that does not exceed 10 minutes in duration [1]. The field of education has witnessed significant advancements in various avenues, and, consequently, this has led to an information overload among students, making them lose attention regarding the process of absorbing knowledge and retaining it. Hence, to make learning more interesting, introducing innovative teaching methods using social media networks will offer great benefits, which will help attract students' attention and engage them in techniques and ways that vary from the traditional teaching method [2]. One such innovative modality is the pitstop method of teaching, which entails using short videos to aid collaborative learning among students and help enhance and improve their learning experience. It facilitates learning and scholarly pursuits by providing opportunities for various types of interactions and discussions, thereby making learning interesting and fun-filled.

Several studies have stated that video lessons that last from one minute to a maximum of three minutes followed by interaction through social platforms and assessment help students enhance their knowledge considerably [2-4]. Short videos have been trending in the entertainment field, and incorporating them into medical education would promote novel ways of teaching [5]. Research has shown that the relative convenience of content generation, as well as creativity, rapid content transmission, and emphasis on sociality, are the distinct attributes of a short video platform [6]. These short videos, when employed as a teaching tool, will help kindle the memory and increase the attention of the students, thereby aiding them to process the information easily. This study aimed to compare the impact of a novel method of teaching, the pitstop method, with that of the standard method of didactic teaching using presentation slides.

## Materials and methods

This comparative cross-sectional study was conducted at a tertiary teaching hospital in India and involved phase II undergraduate medical students. The project proposal was approved by the institutional ethics committee (IRB/IEC Sri Ramachandra Institute of Higher Education and Research, NI/23/AUG/89/85). Relevant permissions were obtained from the dean and heads of the departments.

Phase II undergraduate students were sensitized about the project and 210 out of 250 students who gave consent to participate in the study were included. The other 40 students continued to attend the sessions as per their roll number without providing feedback and their test results were not analyzed. Two sessions comprising a total of 10 videos with five videos in each session were planned for a particular system (a total of 10 sessions). Two types of teaching methodology were used in the videos. For session 1, a short video using a presentation template was used. For session 2, a short video using hand notes and figures was prepared. Each video prepared was two minutes in duration. The other group received a didactic lecture with presentation slides, which is the traditional method used in the institute. Students were divided into two groups according to their roll numbers: A and B batches. For session 1, A batch was the control group and B batch was the test group; for session 2, the test and control groups were reversed. Pre-test and post-test exercises were conducted for each session, including multiple-choice questions, fill-in-the-blanks, and picture-based questions using online resources.

Feedback was obtained from the students regarding the innovative teaching methodology by using a questionnaire, relating to the clarity, innovation, usefulness, and their preference of the teaching method - a video with presentation, a video with handwritten notes and diagrams, or the traditional teaching method. The responses were collected in a feedback questionnaire and ranked on a 5-point Likert scale. A column on open-ended questions on what improves attention during the sessions was also added. Results were analyzed using JASP software. The test marks are presented as mean ±standard deviation (SD) with confidence intervals (CI). Normality was tested using the Shapiro-Wilk test. Paired sample t-test was done for the analysis of pre- and post-test marks. An independent samples t-test was performed to compare marks between groups. Analysis was done by documenting the changes in Likert scale values for A and B batches between pre- and post-sessions.

## Results

In session 1, the test group had a post-test mean of 4.50 ±0.32 compared to the control group's 3.53 ±0.37 (Table 1). In session 2, the test group scored well in the post-test with a mean of 4.43 ±0.38 compared to 3.87 ±0.44 scored by the control group. This difference was statistically significant (p<0.001) (Table 2).

**Table 1:** Comparison of the pre-test and post-test scores between groups (session 1) *Statistically significant CI: confidence interval; SD: standard deviation

Session 1	A (control, n= 95), mean ±SD (CI)	B (test, n=115), mean ±SD (CI)	t	p	Interpretation
Pre-test	3.08 ±0.66 (2.95–3.21)	2.76 ±0.57 (2.65–2.86)	3.82	<0.001*	A>B
Post-test	3.53 ±0.37 (3.46–3.61)	4.50 ±0.32 (4.44–4.55)	-20.06	<0.001*	B>A
p (t value)	<0.001* (-5.89)	<0.001* (-29.25)			

**Table 2:** Comparison of the pre-test and post-test scores between groups (session 2) *Statistically significant CI: confidence interval; SD: standard deviation

Session 2	A (test, n=92), mean ±SD (CI)	B (control, n=112), mean ±SD (CI)	t	p	Interpretation
Pre-test	3.10 ±0.47 (3.01–3.2)	3.05 ±0.64 (2.93–3.17)	0.63	0.53	A=B
Post-test	4.43 ±0.38 (4.35–4.51)	3.87 ±0.44 (3.79–3.95)	9.63	<0.001*	A>B
p (t value)	<0.001 (-21.03)	<0.001 (-11.46)			

Our statistical analysis comparing the difference in teaching tools of both sessions showed that session 2 was comparable to session 1, though the pre-test values were significantly higher in the test group in session 2. Hence, analysis of covariance (ANCOVA) was performed with pre-test value as a covariance. ANCOVA revealed no significant difference between the sessions for the test groups (p=0.74). Feedback from the students (n=210) was analyzed, and 75.2% and 24.8% strongly agreed and agreed, respectively, that the pitstop method of teaching was more interesting than the traditional method of teaching. Of note, 58.58% and 39.52% strongly agreed and agreed (n=210), respectively, that it improved the attention and knowledge retention of the students. More than 70% (n=210) agreed that this newer methodology was very helpful for exam preparation and the content was tailored to the objectives. Notably, 54.2% and 42% strongly agreed and agreed, respectively, that the newer methodology helped in knowledge retention and clinical application (Table 3).

**Table 3:** Feedback from students on the pitstop method

	Strongly agree, n (%)	Agree, n (%)	Don’t know, n (%)	Disagree, n (%)	Strongly disagree, n (%)
Was the teaching methodology interesting?	158 (75.2%)	52 (24.8%)	-	-	-
Was there clarity in the content?	139 (66.2%)	71 (33.8%)	-	-	-
Was the content tailored to meet the objectives of the session?	148 (70.4%)	62 (29.6%)	-	-	-
Was the newer methodology of short videos useful for exam preparation?	161 (76.8 %)	49 (23.2%)	-	-	-
The newer methodology improved my attention	123 (58.58%)	83 (39.52%)	4 (1.9%)	-	-
Did the newer methodology help in knowledge retention and clinical application?	114 (54.2%)	88 (42%)	8 (3.8%)	-	-

**Figure 1:**
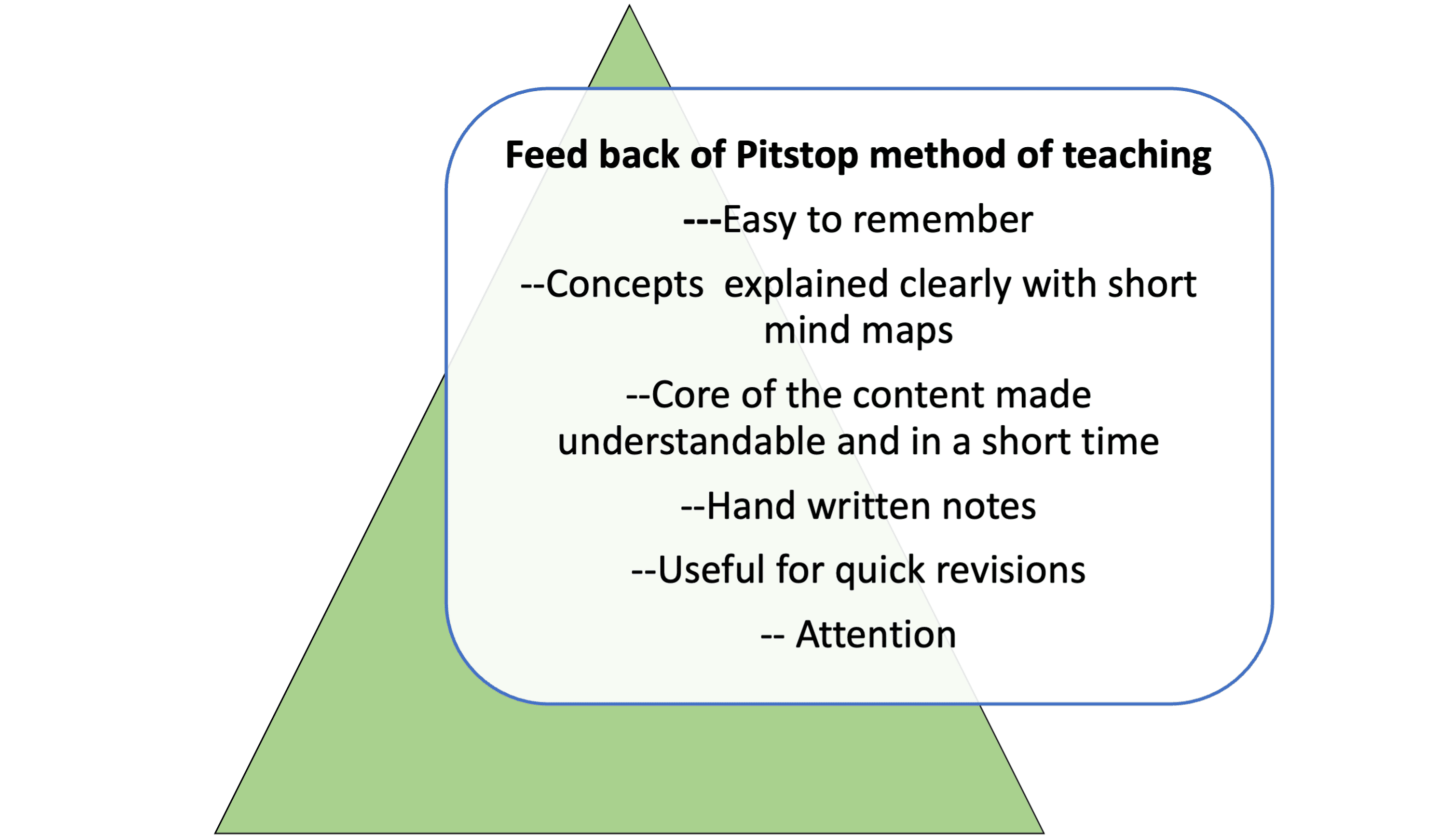
Summary of open-ended feedback on the pitstop method of teaching Author credit: Dr. Subalakshmi Balasubramanian

The study also involved a few open-ended questions on the new method; many students felt that the method improved attention, and written notes given as flashcards were also useful to help recap before the exams, and concepts were explained clearly and could be easily remembered. The majority of the students concurred that the session with video and handwritten notes was more interesting than presentations and didactic lectures (Figure 1).

## Discussion

Our study comparing an innovative method of teaching (pitstop method) with classical teaching has shown that the short-term effects of the tests are better with the pitstop method. The short videos, delivered as either presentations or with handwritten notes, were equally effective. Generation Z will soon be replaced by the alpha generation in academic settings, and these students will be digitally connected in all aspects of their lives, with a wealth of knowledge and information accessible with just a click on their phones [7,8]. Their awareness and knowledge are enhanced by using various online platforms where artificial intelligence and innovation are extensively employed. The students feel that the new method makes learning more fun-filled and knowledge retention is better than the traditional methods of learning, which aligns with several previous studies [9]. As time changes, teachers also should adapt to the newer methodologies of teaching and make the learners engaged rather than just providing traditional methods of teaching. The analysis of first-year medical students’ preferences in slide design revealed the importance of visual appeal, organization, use of diagrams and visuals, consistency in format, and engagement [10].

Teachers play a key and challenging role in this newer methodology. They have to rationally think about the implementation of technology and make the videos interesting by covering all the important aspects within a shorter timeframe [11]. This is quite challenging, but the process of preparing these videos can be rewarding and the teachers can derive a tremendous sense of fulfillment and satisfaction from the excellent outcomes achieved with the implementation of this new method. The implementation of this method will lead to better learning outcomes and knowledge retention than the traditional method of teaching. Though the advancement in technology has many benefits, it is still a double-edged sword, and students face many consequences as well. One of the most worrisome consequences is low attention span and concentration, which can be very unfavorable as good knowledge retention capacity is essential for graduate students.

Studies have shown students still have an affinity for the traditional method, like the chalk-and-talk method, because of its natural pauses and breaks [12]. It also provides time for meaningful interactions, resulting in better concentration, more eye contact, and retention of the class content, with the content better organized in the presentation method [13]. It also helps students build, test, and revise mental models [14]. Chalk talks have been described as a type of bite-sized teaching with the use of visuals [15]. The pitstop is one such method where the traditional method is augmented by using short videos for teaching. The term pitstop is used in formula racing; it is an incredibly fast process where a highly trained crew performs a series of tasks very quickly with utmost precision and concentration, and the same concept can be adopted in the field of education with the pitstop teaching method [16].

In the study by Liu et al. [17], multiple factors were found to affect cognitive development, including the medium of teaching. The others include the ability to absorb knowledge, think critically, and reconstruct meaning. The current generation of students prefers fragmented reading, particularly in its temporal form. Fragmented reading behavior has significant, positive effects on cognitive breadth while it can have a negative impact on depth [17]. To overcome this effect on depth, the pitstop method was added to classical teaching. Attentional fragmentation is referred to as the integration of digital text, pictures, and sound, which stimulate students’ interest through their visual and auditory senses, attracting their attention to a rapid succession of diverse content. We decided to use the pitstop method as this can improve the cognitive effects. "Subitizing" is defined as a phenomenon whereby approximately four items can be quickly and accurately processed. Form perception is a non-numerical cognitive correlate of the relationship between subitizing ability and arithmetic computation [18]. Our videos were short enough to take advantage of this phenomenon.

Content that is fragmented, with the same overall content, results in more working memory capacity, lower working memory load, and less negative impact on cognitive ability compared to unfragmented content. Attention switching between concept and working memory was attributed as the key factor affecting cognitive ability [19]. The audiovisual features of the video grab the attention of the students and help in easy understanding. These short videos kindle the interest of the students in the subject and enjoyment of learning rather than getting stressed out in learning and understanding the various concepts [20]. The use of these short educational videos in our study also proved to afford similar benefits and was compatible with existing literature showing that students performed better and their knowledge retention was good when the pre and post-tests were analyzed by comparing the test and control groups, which were statistically significant.

**Figure 2:**
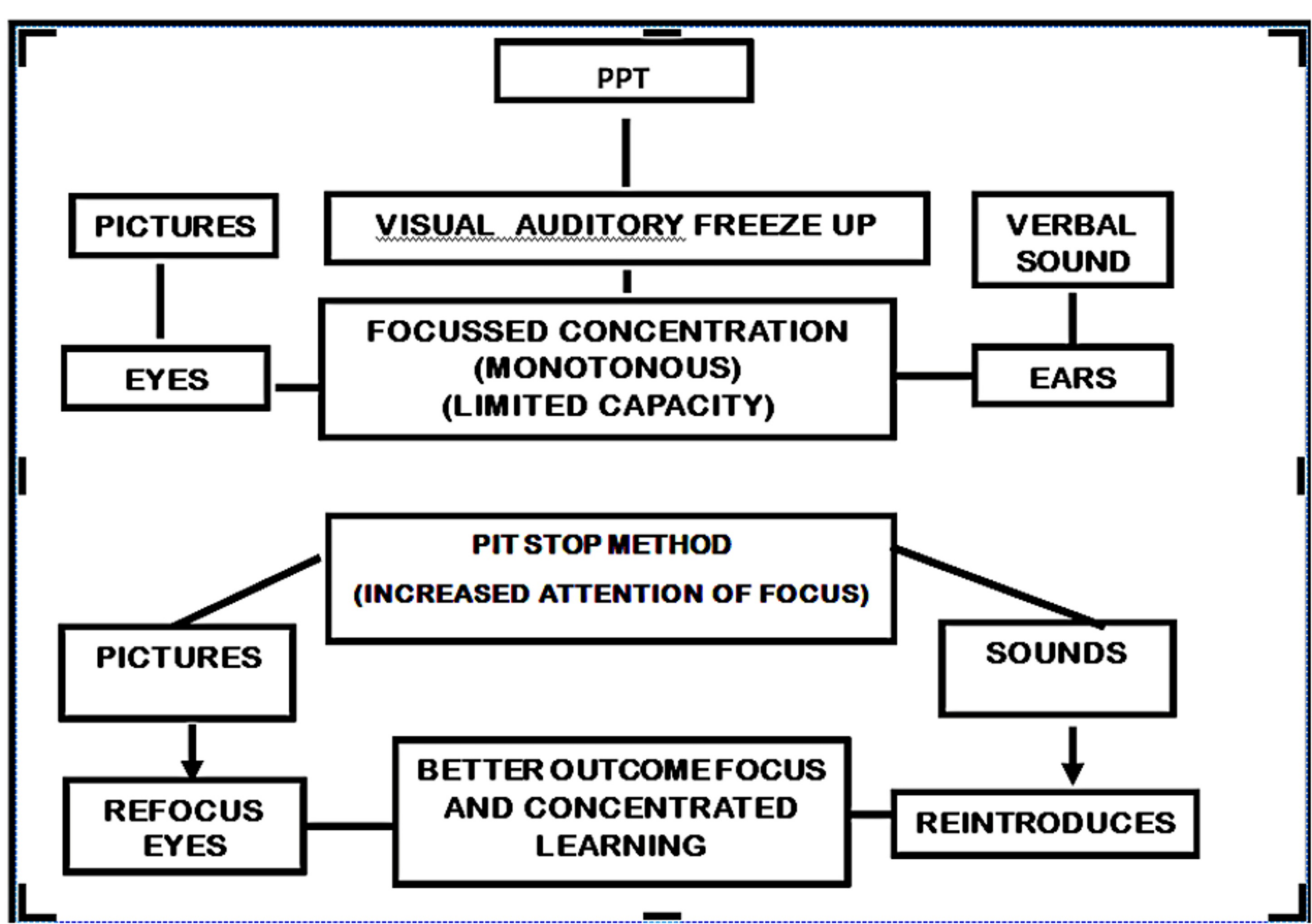
Flowchart highlighting the significance of pitstop method of teaching Author credit: Dr. Nellaiyappan Balasubramanian

Rather than long video clips of 10 minutes or more, short video clips that include innovation like in our study would stimulate both the auditory and visual systems, which would help the students retain the concepts for a longer time and further enhance their interest in the subject. This was statistically proved in our study as well. The video clips also helped the students with quick revision and they could recap the contents at their own pace and this will help them to meet the criteria of Bloom’s Taxonomy (Figure 2).

During a lecture, both the visual and auditory senses are employed to absorb information, and assistance in the form of visual aids is useful in such scenarios [21]. Information processing theory postulates that humans receive information through a sensory registry, process it in short-term memory, and store it for long-term memory. Dual coding theory mentions two channels for receiving and processing information: words and pictures. Cognitive load theory postulates that the load is limited by space, usefulness, and demands on working memory [10].

These results suggest that the movements involved in handwriting enable better memorization of new words. The advantage of handwriting over typing might also be due to a more positive mood during learning [22]. Our study has shown that concerning perception, there is no change between handwritten notes and presentation slides when given through short videos. Conceptual videos can enhance the immediate gain of knowledge and help in consolidating learning from the lectures [23]. Microlearning has been demonstrated to boost confidence in performing procedures and improve knowledge retention in various disciplines within medicine [24]. The pitstop can be regarded as a method of microlearning. Creating short videos that highlight the key objectives is consistent with Peyton’s educational theory for skill learning [25]. It also aligns with the recommendations of keeping the videos brief and targeted, using audio and visual elements to emphasize appropriate parts, and the use of signaling to highlight important concepts [1].

The analysis and feedback from the students revealed that this newer methodology enhanced the learning process, knowledge retention capacity, quick revision, and recollection among the students when compared to the traditional method of teaching.

Limitations of the study

This study has a few limitations. For instance, the students were not categorized based on their type of learning. Moreover, prior performance and long-term effects were not analyzed. Also, qualitative analysis was not performed in this study, which could have revealed the advantages and disadvantages of this method.

## Conclusions

Short videos have become a major trend in the entertainment field, and incorporating them into medical education would provide a novel way of teaching to the current generation of students who are digitally more connected in all walks of life. The pitstop method is one such novel method of teaching, which, when integrated into the curriculum, will have a positive impact on students' attention, knowledge retention, and short-term outcomes. Thoughtfully produced short videos are effective teaching tools and can significantly improve students' performance. This collaborative learning method will make them confident graduates and better physicians with excellent knowledge and skills.

## References

[1] Brame CJ (2016). Effective educational videos: principles and guidelines for maximizing student learning from video content. CBE Life Sci Educ.

[2] Palmon I, Brown CS, Highet A (2021). Microlearning and social media: a novel approach to video-based learning and surgical education. J Grad Med Educ.

[3] Bezzubtseva MV, Demkina AE, Lipilina MN (2022). Video or text? Education through a social media website in hypertension. Int J Cardiol Cardiovasc Risk Prev.

[4] Kim D (2022). Like & share: video-based learning through social media in oral & maxillofacial surgery. J Korean Assoc Oral Maxillofac Surg.

[5] Curran V, Simmons K, Matthews L, Fleet L, Gustafson DL, Fairbridge NA, Xu X (2020). YouTube as an educational resource in medical education: a scoping review. Med Sci Educ.

[6] Zhu J, Yuan H, Zhang Q (2022). The impact of short videos on student performance in an online-flipped college engineering course. Humanit Soc Sci Commun.

[7] Hutajulu JM, Agustiani H, Setiawan AS (2024). Special characteristics of alpha generation children behavior in dentistry: a literature review. Eur J Dent.

[8] Elenga N, Krishnaswamy G (2022). A new generation of physicians-the Generation Z. Are you ready to deal with it?. Front Public Health.

[9] Ang ET, Abu Talib SNB, Thong M, Tze CC (2017). Using video in medical education: what it takes to succeed. Asia Pac Schol.

[10] Bland T, Guo M, Dousay TA (2024). Multimedia design for learner interest and achievement: a visual guide to pharmacology. BMC Med Educ.

[11] Hathur B, Kulkarni P (2024). Changing roles of medical teachers in the era of competency-based medical education. APIK J Intern Med.

[12] Seth V, Upadhyaya P, Ahmad M, Moghe V (2010). PowerPoint or chalk and talk: perceptions of medical students versus dental students in a medical college in India. Adv Med Educ Pract.

[13] Mahanta P, Kalita D, Phukon C (2021). Indian medical undergraduates' perceptions of effective teaching methods: a cross-sectional study. Adv Med Educ Pract.

[14] Singh N, Phoon CK (2021). Not yet a dinosaur: the chalk talk. Adv Physiol Educ.

[15] Martinetti A, Awadhpersad P, Singh S, van Dongen LM (2021). Gone in 2s: a deep dive into perfection analysing the collaborative maintenance pitstop of Formula 1. J Qual Maint Eng.

[16] Hish AJ (2024). A psychiatry clerkship orientation based on bite-sized teaching and chalk talks. Acad Psychiatry.

[17] Liu W, Huang H, Saleem A, Zhao Z (2022). The effects of university students' fragmented reading on cognitive development in the new media age: evidence from Chinese higher education. PeerJ.

[18] Cui Z, Hu Y, Wang X, Li C, Liu Z, Cui Z, Zhou X (2024). Form perception is a cognitive correlate of the relation between subitizing ability and math performance. Cogn Process.

[19] Cao J, Luo J, Zhou J, Jiang Y (2024). Attention switching through text dissimilarity: a cognition research on fragmented reading behavior. Front Hum Neurosci.

[20] Krumm IR, Miles MC, Clay A, Carlos Ii WG, Adamson R (2022). Making effective educational videos for clinical teaching. Chest.

[21] Sahu DR, Supe AN (2000). The art and science of presentation: 35-mm slides. J Postgrad Med.

[22] Ihara AS, Nakajima K, Kake A, Ishimaru K, Osugi K, Naruse Y (2021). Advantage of handwriting over typing on learning words: evidence from an N400 Event-Related Potential Index. Front Hum Neurosci.

[23] Abdulla MH, O'Sullivan E (2019). The impact of supplementing PowerPoint with detailed notes and explanatory videos on student attendance and performance in a physiology module in medicine. Med Sci Educ.

[24] De Gagne JC, Park HK, Hall K, Woodward A, Yamane S, Kim SS (2019). Microlearning in health professions education: scoping review. JMIR Med Educ.

[25] Chand M, Qureshi T (2014). Evolution in surgical training: what can we learn from professional coaches and elite athletes?. J R Soc Med.

